# Role of subcortical structures on cognitive and social function in schizophrenia

**DOI:** 10.1038/s41598-017-18950-2

**Published:** 2018-01-19

**Authors:** Daisuke Koshiyama, Masaki Fukunaga, Naohiro Okada, Fumio Yamashita, Hidenaga Yamamori, Yuka Yasuda, Michiko Fujimoto, Kazutaka Ohi, Haruo Fujino, Yoshiyuki Watanabe, Kiyoto Kasai, Ryota Hashimoto

**Affiliations:** 1 Department of Neuropsychiatry, Graduate School of Medicine, The University of Tokyo, Tokyo, Japan; 2 Division of Cerebral Integration, National Institute for Physiological Sciences, Aichi, Japan; 3 Division of Ultrahigh Field MRI, Institute for Biomedical Sciences, Iwate Medical University, Iwate, Japan; 4 Department of Psychiatry, Osaka University Graduate School of Medicine, Osaka, Japan; 5 Graduate School of Human Sciences, Osaka University, Osaka, Japan; 6 Diagnostic and Interventional Radiology, Osaka University Graduate School of Medicine, Osaka, Japan; 7 Molecular Research Center for Children’s Mental Development, United Graduate School of Child Development, Osaka University, Osaka, Japan; 8 International Research Center for Neurointelligence (WPI-IRCN), The University of Tokyo Institutes for Advanced Study (UTIAS), The University of Tokyo, Tokyo, Japan

## Abstract

Subcortical regions have a pivotal role in cognitive, affective, and social functions in humans, and the structural and functional abnormalities of the regions have been associated with various psychiatric disorders. Although previous studies focused on the neurocognitive and socio-functional consequences of prefrontal and tempolo-limbic abnormalities in psychiatric disorders, those of subcortical structures remain largely unknown. Recently, MRI volume alterations in subcortical structures in patients with schizophrenia have been replicated in large-scale meta-analytic studies. Here we investigated the relationship between volumes of subcortical structures and neurocognitive and socio-functional indices in a large sample of patients with schizophrenia. First, we replicated the results of meta-analyses: the regional volumes of the bilateral hippocampus, amygdala, thalamus and nucleus accumbens were significantly smaller for patients (N = 163) than for healthy controls (HCs, N = 620). Second, in the patient group, the right nucleus accumbens volume was significantly correlated with the Digit Symbol Coding score, which is known as a distinctively characteristic index of cognitive deficits in schizophrenia. Furthermore, the right thalamic volume was significantly correlated with social function scores. In HCs, no significant correlation was found. The results from this large-scale investigation shed light upon the role of specific subcortical nuclei on cognitive and social functioning in schizophrenia.

## Introduction

Subcortical structures have important roles on exerting cognitive, affective, and social functions in humans^1–6^. The structural and functional abnormalities of the regions have been associated with various psychiatric disorders including schizophrenia, depression, and autism spectrum disorders^7–16^. However, it is largely unknown how abnormalities of specific subcortical nuclei are associated with neurocognitive and socio-functional consequences. Altered volumes in subcortical structures such as the basal ganglia and thalamus have been pointed out in schizophrenia^7,17–22^. Recent large-scale multicenter studies have been conducted such as the Enhancing Neuro Imaging Genetics through Meta-Analysis (ENIGMA) Consortium^12,14,16,23–27^. The ENIGMA Schizophrenia Working Group (ENIGMA-SZ)^15^ revealed that the volumes of hippocampus, amygdala, thalamus, and nucleus accumbens (NA) were smaller and the volumes of caudate, putamen, and globus pallidus were larger in patients with schizophrenia than in healthy controls (HCs). Our research group also performed a multicenter study, and not only replicated the results of ENIGMA-SZ, but also showed left-hemisphere-biased volume alteration in globus pallidus^13^. However, cognitive consequences of altered volumes of subcortical structures in patients with schizophrenia remains to be investigated.

Several prior studies evaluated the association between subcortical regional volume and cognitive function in patients with schizophrenia, but they are limited to temporolimbic structures (hippocampus and amygdala)^28–34^. Kubota *et al*.^35^ also revealed that lateral ventricular volumes were associated with intelligent quotient (IQ) in patients with schizophrenia. However, the relationship between subcortical regional volumes and specific cognitive functions has not been clarified in patients with schizophrenia.

Furthermore, the influence of volume reductions in subcortical structures upon real-world social functioning has also been unclear. According to a clinical study, patients with unilateral thalamic infarction had impaired social cognition^36^. Patients with schizophrenia show severe impairment of social function and have difficulty in social daily life^37–40^. Volume reduction of subcortical structures such as the thalamus might affect social functioning. However, to the author’s knowledge, there has been no prior large-scale study that focused on the relationship between subcortical regional volumes and social functioning in patients with schizophrenia.

In our current study, we sought to observe the relationship between subcortical regional volumes [basal ganglia, thalamus, and temporolimbic structures (hippocampus and amygdala)] and cognitive/social function in patients with schizophrenia. We particularly focused on the Full-Scale IQ (FIQ) and Digit Symbol Coding subscales of the Wechsler Adult Intelligence Scale-Third Edition (WAIS-III)^41^ for the assessment of cognitive function, and the Comprehension and Picture Arrangement subscales of the WAIS-III, and the University of California, San Diego, Performance-Based Skills Assessment Brief (UPSA-B)^42^ for the assessment of social function. The FIQ represents global cognitive function, and the Digit Symbol Coding score is the most influential factor in identifying cognitive deficits in schizophrenia patients^43–46^. Previous studies showed that the Comprehension and Picture Arrangement scores reflect social knowledge and perception^47,48^, and the UPSA-B measures functional capacity for everyday life. For the supplementary analyses, we investigated the correlations between those subcortical regional volumes and the other subscales of the WAIS-III.

## Results

### Demographics

The demographic and clinical characteristics of patients with schizophrenia and HCs are shown in Table 1. Patients and HCs did not differ significantly in age or gender. Histograms of the age distribution in both groups are shown in Supplementary Figure 1. Patients and HCs differed in the years of education (patients with schizophrenia: mean = 14.0, SD = 2.6; HCs: mean = 15.0, SD = 2.1; *p* = 1.6 × 10^−7^) and premorbid IQ (patients with schizophrenia: mean = 102.0, SD = 10.4; HCs: mean = 108.4, SD = 7.7; *p* = 1.8 × 10^−17^).

**Table 1:** Demographic and clinical characteristics of participants.

	SZ (N = 163)		HC (N = 620)		Statistics		
	Mean	SD	Mean	SD	Effect size, *d*	*t* or *χ*^2^	*p*
Age	35.0 (16–71)	11.6	34.1 (18–66)	13.0	0.07	*t* (781) = −0.80	0.42
Male/Female	91/72		305/315			χ^2^ (1.0) = 2.27	0.13
Education (years)	14.0	2.6	15.0	2.1	−0.44	*t* (781) = 5.29	1.6 × 10 ^−7^
Premorbid IQ^a^	102.0	10.4	108.4	7.7	−0.71	*t* (773) = 8.72	1.8 × 10 ^−17^
Duration of illness (years)	11.2	9.0					
PANSS Positive^b^	18.9	5.6					
PANSS Negative^b^	19.9	5.6					
PANSS General^b^	43.3	10.5					
PANSS Total^b^	82.1	19.6					
CPZ equivalent (mg/day)	594.1	540.3					

^a^Seven patients with schizophrenia and one HC have no premorbid IQ data; ^b^Two patients with schizophrenia have no PANSS data. Underlining indicates *p* < 0.05. Age-span was shown in the brackets nearby mean age. Abbreviation: SZ, schizophrenia; HC, healthy control; SD, standard deviation; IQ, intelligence quotient; PANSS, positive and negative syndrome scale; CPZ, chlorpromazine.

### MRI indices

**Figure 1:**
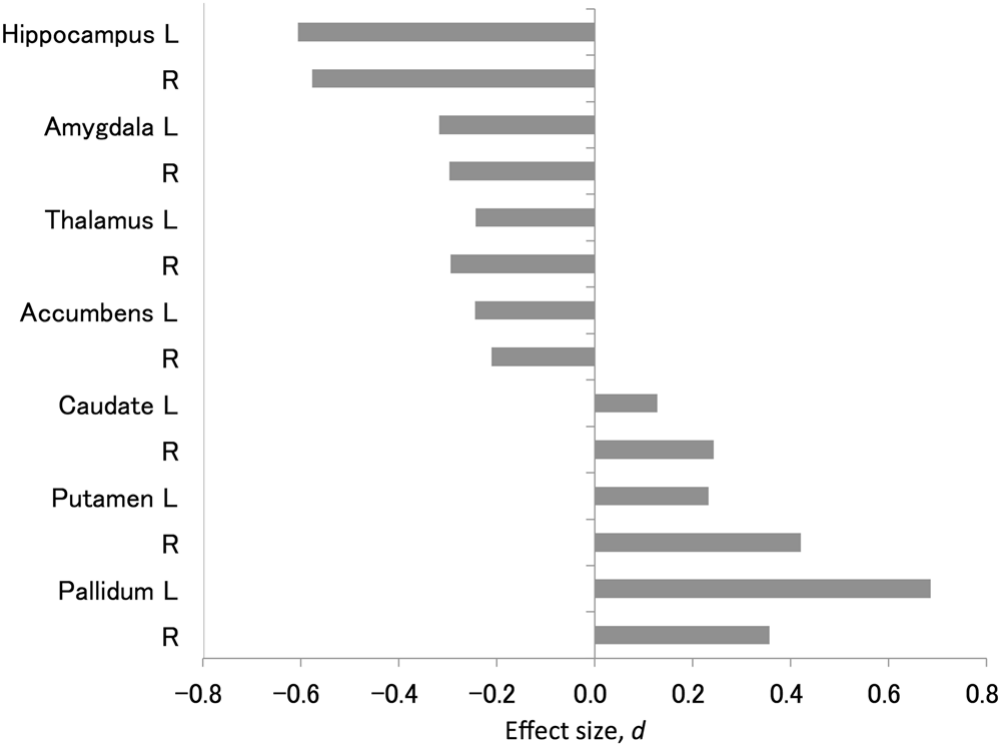
Cohen’s *d* effect sizes for the difference in MRI indices between patients with schizophrenia and HCs. The vertical axis represents the MRI indices. The horizontal axis represents Cohen’s *d* effect sizes for the difference in MRI indices between patients with schizophrenia and HCs. Abbreviation: L, left; R, right.

The normalized regional volumes of the bilateral hippocampus (left: *d* = −0.61, *p* = 6.0 × 10^−13^; right: *d* = −0.58, *p* = 7.2 × 10^−12^), amygdala (left: *d* = −0.32, *p* = 3.8 × 10^−4^; right: *d* = −0.30, *p* = 6.2 × 10^−4^), thalamus (left: *d* = −0.24, *p* = 4.5 × 10^−3^; right: *d* = −0.29, *p* = 6.5 × 10^−4^) and NA (left: *d* = −0.24, *p* = 5.4 × 10^−3^; right: *d* = −0.21, *p* = 1.3 × 10^−2^) were significantly smaller for patients with schizophrenia than for HCs (Table 2, Fig. 1). In contrast, the normalized regional volumes of right caudate (*d* = 0.24, *p* = 5.9 × 10^−3^), bilateral putamen (left: *d* = 0.23, *p* = 7.9 × 10^−3^; right: *d* = 0.42, *p* = 1.8 × 10^−6^), and bilateral pallidum (left: *d* = 0.69, *p* = 4.4 × 10^−15^; right: *d* = 0.36, *p* = 6.0 × 10^−5^) were significantly larger for patients with schizophrenia than for HCs (Table 2, Fig. 1). The correlation maps among the regional brain volumes are shown in Supplementary Figure 2 for all participants, Supplementary Figure 3 for schizophrenia group, and Supplementary Figure 4 for HCs.

**Table 2:** Comparison of the MRI indices and cognitive/social indices of interest between patients with schizophrenia and HCs.

		SZ (N = 163)		HC (N = 620)		Statistics		
		Mean	SD	Mean	SD	Effect size, *d*	*t* or *χ*^2^	*p*
MRI indices								
Hippocampus	L	2553.8	415.4	2783.6	339.3	−0.61	*t* (781) = 7.32	6.0 × 10 ^−13^
	R	2049.9	393.8	2258.1	324.1	−0.58	*t* (781) = 6.96	7.2 × 10 ^−12^
Amygdala	L	811.8	155.8	862.2	161.8	−0.32	*t* (781) = 3.57	3.8 × 10 ^−4^
	R	987.9	198.4	1045.1	186.6	−0.30	*t* (781) = 3.44	6.2 × 10 ^−4^
Thalamus	L	2060.3	738.9	2232.0	670.5	−0.24	*t* (781) = 2.85	4.5 × 10 ^−3^
	R	1900.6	594.8	2070.0	553.1	−0.29	*t* (781) = 3.42	6.5 × 10 ^−4^
Accumbens	L	876.6	97.3	900.4	96.7	−0.24	*t* (781) = 2.79	5.4 × 10 ^−3^
	R	651.7	93.8	670.3	82.3	−0.21	*t* (781) = 2.49	1.3 × 10 ^−2^
Caudate	L	2039.4	365.8	1991.5	381.5	0.13	*t* (781) = −1.44	0.15
	R	2753.0	398.8	2656.0	399.7	0.24	*t* (781) = −2.76	5.9 × 10 ^−3^
Putamen	L	5894.3	594.7	5759.0	572.7	0.23	*t* (781) = −2.66	7.9 × 10 ^−3^
	R	5957.9	519.8	5742.7	504.8	0.42	*t* (781) = −4.81	1.8 × 10 ^−6^
Pallidum	L	1383.9	221.4	1238.2	202.8	0.69	*t* (781) = −8.00	4.4 × 10 ^−15^
	R	1205.8	171.9	1144.3	173.5	0.36	*t* (781) = −4.03	6.0 × 10 ^−5^
Cognitive indices of interest								
Full-Scale IQ		88.1	17.9	112.0	12.1	−1.57	*t* (781) = 20.12	7.3 × 10 ^−73^
Digit Symbol-Coding		6.5	3.0	11.8	3.0	−1.79	*t* (781) = 20.44	1.0 × 10 ^−74^
Social indices of interest								
Comprehension		7.7	3.5	12.8	2.7	−1.65	*t* (781) = 20.39	2.1 × 10 ^−74^
Picture Arrangement		7.9	3.9	10.6	3.2	−0.75	*t* (781) = 9.02	1.4 × 10 ^−18^
Total score of the UPSA-B^a^		67.8	16.8	82.9	7.8	−1.16	*t* (217) = 9.14	4.6 × 10 ^−17^

^a^Only 68 patients with schizophrenia and 151 HCs have the UPSA-B data. The unit of MRI indices is mm^3^. Underlining indicates *p* < 0.05. Abbreviation: SZ, patients with schizophrenia; HCs, healthy controls; SD, standard deviation; MRI, magnetic resonance imaging; L, left; R, right; IQ, intelligence quotient; UPSA-B, the University of California, San Diego, Performance-Based Skills Assessment Brief.

### Cognitive and social indices of interest

**Figure 2:**
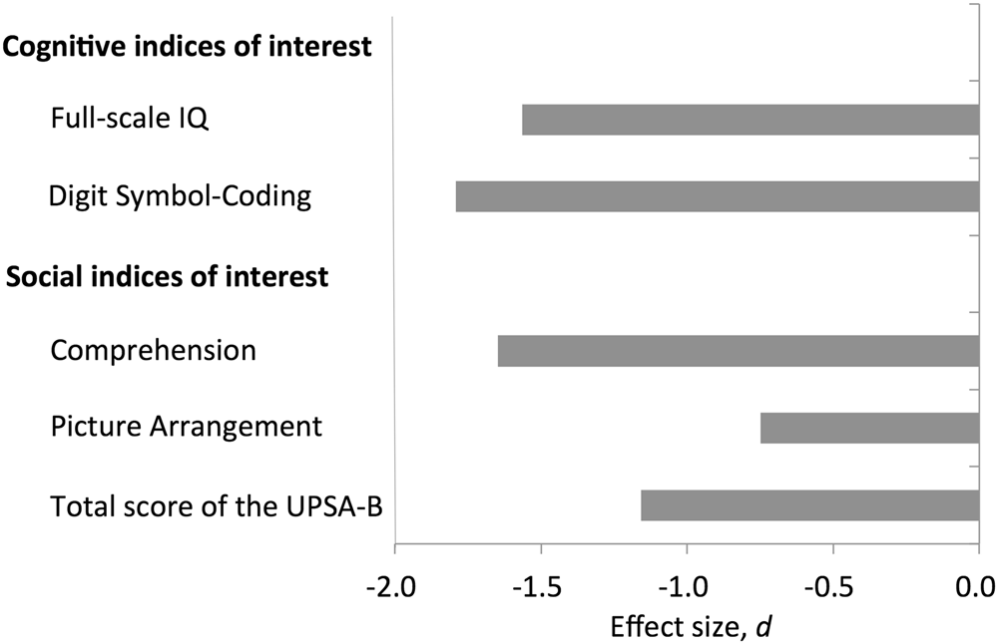
Cohen’s *d* effect sizes for the difference in cognitive/social indices of interest between patients with schizophrenia and HCs. The vertical axis represents cognitive/social indices of interest. The horizontal axis represents Cohen’s *d* effect sizes for the difference in cognitive/social indices of interest between patients with schizophrenia and HCs. Abbreviation: IQ, intelligence quotient; UPSA-B, the University of California, San Diego, Performance-Based Skills Assessment Brief.

All neuropsychological measures were lower for patients than for HCs (all *p* < 0.05; Table 2, Fig. 2, Supplementary Table 1). FIQ (*d* = −1.57, *p* = 7.3 × 10^−73^) was severely decreased in patients relative to HCs. The Digit Symbol Coding score (*d* = −1.79, *p* = 1.0 × 10^−74^) was the most severe affected in patients with schizophrenia compared to HCs. The Comprehension (*d* = −1.65, *p* = 2.1 × 10^−74^), Picture Arrangement (*d* = −0.75, *p* = 1.4 × 10^−18^), and the total score of the UPSA-B were also impaired in patients compared with HCs (*d* = −1.16, *p* = 4.6 × 10^−17^). The correlation maps among the premorbid IQ and cognitive and social indices are shown in Supplementary Figure 5 for all participants, Supplementary Figure 6 for schizophrenia group, and Supplementary Figure 7 for HCs.

### Correlation analysis

We examined the correlation between the MRI indices and cognitive/social indices of interest in patients with schizophrenia. In patients with schizophrenia, gray matter volumes in bilateral hippocampus (lt: *r* = 0.301, *p* = 9.6 × 10^−5^; rt: *r* = 0.301, *p* = 9.4 × 10^−5^), left amygdala (*r* = 0.293, *p* = 1.5 × 10^−4^), right thalamus (*r* = 0.299, *p* = 1.0 × 10^−4^) and right NA volume (*r* = 0.267, *p* = 5.6 × 10^−4^) were correlated with FIQ. The right NA volume (*r* = 0.266, *p* = 6.0 × 10^−4^) was correlated with scores on the Digit Symbol Coding. Volumes in right thalamus (*r* = 0.296, *p* = 1.3 × 10^−4^) were correlated with the Comprehension scores; volumes in right hippocampus (*r* = 0.265, *p* = 6.3 × 10^−4^) and right thalamus (*r* = 0.297, *p* = 1.2 × 10^−4^) were correlated with the Picture Arrangement. Right thalamic volume was also associated with total scores on the UPSA-B (*r* = 0.452, *p* = 1.1 × 10^−4^). These results are shown in Table 3. Correlations between the MRI indices and the other indices of WAIS-III or the UPSA-B are listed in Supplementary Tables 2 and 3 for the schizophrenia group and HCs. In HCs, no significant correlation was found.

**Table 3:** Correlations and partial correlations with adjustment for medication between the MRI indices and cognitive/social indices of interest in patients with schizophrenia.

		Cognitive indices of interest				Social indices of interest					
		Full-Scale IQ		Digit Symbol-Coding		Comprehension		Picture Arrangement		Total score of the UPSA	
		*r*	*p*	*r*	*p*	*r*	*p*	*r*	*p*	*r*	*p*
Hippocampus	L	0.301	9.6 × 10 ^−5^	0.211	6.9 × 10^−3^	0.230	3.1 × 10^−3^	0.175	2.6 × 10^−2^	0.247	4.3 × 10^−2^
		(0.326)	(7.1 × 10^−3^)								
	R	0.301	9.4 × 10 ^−5^	0.221	4.6 × 10^−3^	0.216	5.6 × 10^−3^	0.265	6.3 × 10 ^−4^	0.323	7.2 × 10^−3^
		(0.441)	(1.9 × 10^−4^)								
Amygdala	L	0.293	1.5 × 10 ^−4^	0.177	2.4 × 10^−2^	0.230	3.2 × 10^−3^	0.220	4.8 × 10^−3^	0.211	8.4 × 10^−2^
		(0.370)	(2.0 × 10^−3^)								
Thalamus	R	0.299	1.0 × 10 ^−4^	0.219	5.0 × 10^−3^	0.296	1.3 × 10 ^−4^	0.297	1.2 × 10 ^−4^	0.452	1.1 × 10 ^−4^
		(0.412)	(5.3 × 10^−4^)			(0.380)	(1.5 × 10^−3^)	(0.431)	(2.7 × 10^−4^)		
Accumbens	R	0.267	5.6 × 10 ^−4^	0.266	6.0 × 10 ^−4^	0.209	7.4 × 10^−3^	0.131	9.7 × 10^−2^	0.251	3.9 × 10^−2^
		(0.342)	(4.6 × 10^−3^)	(0.231)	(6.0 × 10^−2^)						

Upper line shows correlations between the MRI indices and cognitive/social indices of interest in patients with schizophrenia. Lower line shows partial correlation between the MRI indices and cognitive/social indices of interest with adjusting for chlorpromazine equivalents for main findings (gray marker of upper line) in patients with schizophrenia. Underlining indicates *p* < 1.79 × 10^−3^ (0.05/28) for upper line. Underlining indicates *p* < 0.05 for lower line. Abbreviations: IQ, intelligence quotient; UPSA-B, the University of California, San Diego, Performance-Based Skills Assessment Brief; L, left; R, right.

For the statistical considerations of the medication effect on the main findings, indices of the FIQ (*r* = −0.275, *p* = 3.9 × 10^−4^), Digit Symbol Coding (*r* = −0.192, *p* = 1.4 × 10^−2^), Comprehension (*r* = −0.165, *p* = 3.5 × 10^−2^) and Picture Arrangement (*r* = −0.212, *p* = 6.5 × 10^−3^) were significantly correlated with the chlorpromazine equivalents in the schizophrenia group. The total score of the UPSA-B and MRI indices did not correlate significantly with the chlorpromazine equivalents with the exception of the bilateral caudate (lt: *r* = 0.187, *p* = 1.7 × 10^−2^; rt: *r* = 0.235, *p* = 2.6 × 10^−3^) and pallidum (lt: *r* = 0.201, *p* = 1.0 × 10^−2^; rt: *r* = 0.189, *p* = 1.6 × 10^−2^) in the schizophrenia group. We performed partial correlational analysis adjusted for the chlorpromazine equivalents between the MRI and cognitive/social indices of interest in the schizophrenia group; the correlations for the main findings remained significant (Table 3).

The difference between the groups in the volume-FIQ correlation coefficients was significant for the left hippocampus (z = 3.02, *p* = 2.6 × 10^−3^), the right hippocampus (z = 3.06, *p* = 2.2 × 10^−3^), and the left amygdala (z = 3.04, *p* = 2.4 × 10^−3^). The group difference did not reach significance for the right thalamus or right NA [significance threshold was set at *p* < 1.0 × 10^−2^ (0.05/5) because the FIQ was significantly correlated with five volumes]. The group difference was significant in the correlation coefficients between the right NA volume and the Digit Symbol Coding (z = 2.63, *p* = 8.5 × 10^−3^; significance threshold was set at *p* < 0.05 because the Digit Symbol Coding was significantly correlated with only one volume). That was also true for the right thalamic volume and the Comprehension (z = 3.30, *p* = 9.8 × 10^−4^; significance threshold was set at *p* < 0.05 because Comprehension was significantly correlated with only one volume), Picture Arrangement [z = 2.86, *p* = 4.3 × 10^−3^; significance threshold was set at *p* < 2.5 × 10^−2^ (0.05/2) because Picture Arrangement was significantly correlated with two volumes], and UPSA-B (z = 3.52, *p* = 4.3 × 10^−4^; significance threshold was set at *p* < 0.05 because UPSA-B was significantly correlated with only one volume).

## Discussion

The current study observed the following findings: the normalized regional volumes of the bilateral hippocampus, amygdala, thalamus and NA were significantly smaller in patients with schizophrenia than in HCs; the volumes of the right caudate, bilateral putamen, and bilateral pallidum were significantly larger in patients than in HCs. In patients only, volumes of bilateral hippocampus, left amygdala, right thalamus and right NA were correlated with FIQ; the right NA volume with the Digit Symbol Coding; the right thalamic volume with Comprehension, Picture Arrangement, and UPSA-B. In HCs, no significant correlations were found. Furthermore, the results remained almost the same even after adjusting for medication effects.

We replicated the results of previous meta-analyses^13,15^: the volume reductions of the hippocampus, amygdala, thalamus and NA, and the volume enlargement of the caudate, putamen, and pallidum in patients with schizophrenia. Previous studies pointed out medication effects on volumes of the basal ganglia^49,50^. Although our results showed significant positive correlations of medications with the caudate and pallidum volumes in patients with schizophrenia, those previous studies showed inconsistent results for medication effects on caudate and pallidum volumes. Thus, the medication effect on volumes of the basal ganglia remains controversial in schizophrenia.

Volumes of the hippocampus, amygdala, thalamus, and NA were associated with global cognitive function as assessed by FIQ. Furthermore, these correlations of the hippocampus and amygdala were specific for the schizophrenia group. These findings revealed that the volume reductions of the temporolimbic structures (hippocampus and amygdala) generally affect cognitive function in patients with schizophrenia.

In the current study, the largest patient-control effect size (*d* = −1.79) was found for the Digit Symbol Coding among all subtests of the WAIS-III. The finding agrees with a previous meta-analysis study by Dickinson *et al*.^44^ (*d* = −1.57). Our study observed that the NA volume was correlated with the Digit Symbol Coding in patients with schizophrenia. The function of the NA in the reward system is well-known^5^. Fervaha *et al*.^51^ found a positive association between intrinsic motivation and cognitive test performance in patients with schizophrenia; they suggested that test performance is not purely a measure of ability. When patients with schizophrenia perform cognitive tests, especially severely affected neurocognitive tasks such as the Digit Symbol Coding, motivation might be necessary to be driven by the NA.

All social indices of interest were positively correlated with right thalamic volume in patients with schizophrenia. Andreasen *et al*.^52–54^ proposed that dysfunction in the fronto-thalamic-cerebellar circuitry affected cognitive dysfunction in schizophrenia. In prior positron-emission tomography studies, patients with schizophrenia who were given memory tasks or theory of mind tasks showed lower blood flow in the prefrontal cortex, thalamus and cerebellum compared to HCs^55,56^. Moreover, Browning *et al*.^57^ showed that the thalamus contributes to cognition such as learning, memory and decision-making via interactions with the prefrontal cortex in rhesus monkeys using a disconnection lesion approach. We suggest that right thalamic volume reduction affects social function by disturbing interactions with other regions including the prefrontal cortex in patients with schizophrenia.

There are some limitations to our study. First, handedness was not evaluated in the current correlation analysis. Second, the current study was a cross-sectional study; thus, a causal relationship between cognitive dysfunction and brain regional volume reduction cannot be drawn. Further longitudinal research will be required to elucidate this limitation.

In conclusion, while previous schizophrenia research focused on prefrontal and temporo-limbic structures as the basis for cognitive and social dysfunction in schizophrenia, the importance of this large-scale investigation is the discovery of the association between regional volumes in specific subcortical nuclei and cognitive and social functioning. The next step will be to investigate the causal relationship between cortical-subcortical circuitry and cognitive/social consequences by translating between animal and human studies with an aim toward ultimately developing circuit-based intervention strategies in schizophrenia treatment.

## Methods

### Subjects

One hundred sixty-three patients with schizophrenia and 620 HCs participated in the current study (Table 1). Subjects had participated in previous behavioral and neuroimaging studies^13,58–62^. The subjects were excluded if they had neurological or medical conditions that could potentially affect the central nervous system, such as atypical headache, head trauma with loss of consciousness, chronic lung disease, kidney disease, chronic hepatic disease, thyroid disease, active cancer, cerebrovascular disease, epilepsy, seizures, substance-related disorders or mental retardation. We recruited patients with schizophrenia from outpatient and inpatient units at Osaka University Hospital. Each patient was diagnosed by at least two trained psychiatrists according to the criteria given in the Diagnostic and Statistical Manual of Mental Disorders, Fourth Edition (DSM-IV) based on the Structured Clinical Interview for DSM-IV (SCID). The HCs were recruited through local advertisements at Osaka University. Psychiatrically, medically and neurologically, the HCs were evaluated using the non-patient version of the SCID to exclude individuals who had current or past contact with psychiatric services or who had received psychiatric medication. Estimated premorbid IQ was assessed with the Japanese version of the National Adult Reading Test^63^. Psychotic symptoms were evaluated using the Positive and Negative Syndrome Scale (PANSS)^64^. Patient medication dosage was converted to chlorpromazine (CPZ) equivalents^65^. Written informed consent was obtained from each subject before participation. The current study was approved by the Research Ethical Committee of Osaka University and the Ethical Committee of the Faculty of Medicine, the University of Tokyo, and was conducted in accordance with the Declaration of Helsinki.

### Image analysis

We performed MRI scanning and obtained T1-weighted images with two machines: Osaka A and Osaka B. We scanned 100 patients and 384 HCs with Osaka A, and 63 patients and 236 HCs with Osaka B. The scanner type was a GE 1.5 T, Signa EXCITE for Osaka A. T1-weighted images, using a fast spoiled gradient echo (SPGR) and a head QD coil, were acquired with the following parameters: repetition time (TR) = 12.6 ms, echo time (TE) = 4.2 ms, inversion time (TI) = 400 ms, flip angle = 15 degrees, matrix = 256 × 256 × 124, field of view (FOV) = 240 × 240 × 172 mm, voxel size = 0.9375 × 0.9375 × 1.4 mm, slice thickness = 1.4 mm, number of slices = 124. The slice orientation was in the sagittal plane. The scanner type was a GE 3.0 T, Signa HDxt for Osaka B. T1-weighted images, using a fast SPGR and an 8HRBRAIN coil, were acquired with the following parameters: TR = 7.2 ms, TE = 2.9 ms, TI = 400 ms, flip angle = 11 degrees, matrix = 256 × 256 × 172, FOV = 240 × 240 × 172 mm, voxel size = 0.9375 × 0.9375 × 1 mm, slice thickness = 1 mm, number of slices = 172. The slice orientation was in the sagittal plane.

We performed image processing in the same way as that performed in our previous study^13^. We checked original T1-weighted images through visual inspection for the quality control. We excluded images with a low signal-to-noise ratio or any artifacts, those with partial deficits, and those with any organic abnormal findings. Next, we processed T1-weighted imaging data that had passed the first quality control step with FreeSurfer software version 5.3 (http://surfer.nmr.mgh.harvard.edu)^66^. Through this procedure, we obtained images showing the subcortical segmentation and regional volumes [for the hippocampus, amygdala, thalamus, NA, caudate, putamen, globus pallidus on the both sides and the intracranial volume (ICV)]. After that, two independent researchers visually inspected each segmentation image to exclude images with poor parcellation. No subject was excluded owing to the failure of FreeSurfer processing. After the two quality control steps, we obtained the raw subcortical volume data. The analytical methods used in the study by van Erp *et al*.^15^ from ENIGMA-SZ were followed in this analysis.

We employed the normalized regional volume to remove the effects of the confounding factors that considered linear and nonlinear age effects on subcortical regional volumes (scatter plots between raw subcortical brain volumes and age are shown in Supplementary Figure 8 for all participants, Supplementary Figure 9 for the schizophrenia group, and Supplementary Figure 10 for HCs). We firstly performed a linear regression with the following formula:

$${\rm{Raw}}\,{\rm{volume}}={{\rm{\beta }}}_{{\rm{1}}}\times {\rm{age}}+{{\rm{\beta }}}_{{\rm{2}}}\times {{\rm{age}}}^{{\rm{2}}}+{{\rm{\beta }}}_{{\rm{3}}}\times {\rm{sex}}+{{\rm{\beta }}}_{{\rm{4}}}\times {\rm{ICV}}+{{\rm{\beta }}}_{{\rm{5}}}\times {\rm{machine}}+{\rm{\varepsilon }}$$

Dummy variables were created for sex (male = 1, female = 2) and for the machine (Osaka A = 1, Osaka B = 2). ε denotes the normalized volume, and the normalized volume was derived from the equation above. We used these normalized regional volumes (for the hippocampus, amygdala, thalamus, NA, caudate, putamen and globus pallidus on both sides) as the MRI indices in statistical analysis.

### Neuropsychological measures

We measured subscales of the WAIS-III^41^ and the UPSA-B^42^ to assess cognitive and social function in patients with schizophrenia and HCs. We used FIQ and the Digit Symbol Coding subscale as cognitive indices of interest. The Comprehension and Picture Arrangement subscales, and total scores of the UPSA-B were used as social indices of interest. The raw scores of the WAIS-III were converted to scaled scores to normalize for age. The UPSA-B was developed as an abbreviated version of the UPSA to assess functional daily living skills of patients in a role-play setting, with scores based on performing tasks related to finance (e.g., counting money) and communication (e.g., dialing a number from memory and rescheduling a doctor’s appointment)^42,67^. Scores range from 0–100, with higher scores indicating greater ability in everyday activities.

### Statistical analysis

All statistical analyses were conducted using SPSS (version 23.0.0.0, IBM Corp., Armonk, NY). For comparison of demographic data between patients and controls, we used a significance threshold of *p* < 0.05 for *t*-tests and *χ*^2^ tests. For each demographics, Cohen’s *d* effect sizes were calculated from the overall group contrast.

For comparison of the MRI indices and neuropsychological indices between patients and HCs, we used a significance threshold of *p* < 0.05 for *t*-tests. Since previous studies including meta-analysis have found robust abnormalities in these indices^13^, and tests for group difference was not our primary objective, we did not use the Bonferroni correction here. Rather, for each MRI and neuropsychological index, Cohen’s *d* effect sizes were calculated from the overall group contrast. Furthermore, we showed the Pearson correlation coefficients (*r*) map among regional brain volumes and of the premorbid IQ and cognitive and social indices to show the relationships both within and across groups, respectively.

To demonstrate the relationship between the MRI indices and neuropsychological indices, we calculated the Pearson correlations coefficient (*r*). Correlation models were independently examined for each of the 14 anatomical regions within both groups (the schizophrenia group and the HC group), and therefore a *p*-value of <1.79 × 10^−3^ (0.05/28) was considered statistically significant according to the Bonferroni correction. We did not consider correction for the five neuropsychological indices (two cognitive indices of interest and three social indices of interest) because we did not intend to compare volume-neurocognition associations among regions but sought to examine relationships between regional volumes and each single neurocognitive index of interest. On the other hand, we considered corrections for 14 anatomical regions since we sought to determine which region would show correlations with each neurocognitive index.

Then, we sought to partial out the effect of medication on our main finding of associations between the MRI indices and neuropsychological indices in the schizophrenia group. We first calculated Pearson’s *r* between the chlorpromazine equivalents and the MRI indices and neuropsychological indices. A *p*-value of <0.05 was considered statistically significant; Bonferroni correction was not used here because we sought to sensitively pick up potential confounds. If there was a significant correlation with chlorpromazine equivalents, we then calculated the partial correlation, adjusting for medication in each combination of the MRI and neuropsychological indices.

Furthermore, if we found a significant correlation between the MRI indices and neuropsychological indices in the schizophrenia group, we further tested whether the correlation was specific to schizophrenia by comparing Fisher’s *r*-to-*z* transformed correlational coefficients between the schizophrenia and control groups. Bonferroni correction was applied when appropriate and a corrected *p*-value of <0.05 was considered statistically significant.

## Electronic supplementary material

Supplementary Information
