# Supplementary material for: Role of subcortical structures on cognitive and social function in schizophrenia
Source: Sci Rep. 2018 Jan 19;8:1183. doi: 10.1038/s41598-017-18950-2 (PMC5775279; doi:10.1038/s41598-017-18950-2)

Role of subcortical structures on cognitive and social function in schizophrenia

Daisuke Koshiyama, M.D.[1], Masaki Fukunaga, Ph.D.[2], Naohiro Okada, M.D., Ph.D.[1], Fumio Yamashita, Ph.D.[3], Hidenaga Yamamori, M.D., Ph.D.[4], Yuka Yasuda, M.D., Ph.D.[4], Michiko Fujimoto, M.D., Ph.D.[4], Kazutaka Ohi, M.D., Ph.D.[4], Haruo Fujino, Ph.D.[5], Yoshiyuki Watanabe M.D., Ph.D.[6], Kiyoto Kasai M.D., Ph.D.[1*], Ryota Hashimoto M.D., Ph.D.[4, 7]

1. Department of Neuropsychiatry, Graduate School of Medicine, The University of Tokyo, Tokyo, Japan
2. Division of Cerebral Integration, National Institute for Physiological Sciences, Aichi, Japan
3. Division of Ultrahigh Field MRI, Institute for Biomedical Sciences, Iwate Medical University, Iwate, Japan
4. Department of Psychiatry, Osaka University Graduate School of Medicine, Osaka, Japan
5. Graduate School of Human Sciences, Osaka University, Osaka, Japan
6. Diagnostic and Interventional Radiology, Osaka University Graduate School of Medicine, Osaka, Japan
7. Molecular Research Center for Children's Mental Development, United Graduate School of Child Development, Osaka University, Osaka, Japan

*Corresponding Author

Supplementary Table 1 Comparison of the WAIS-III and UPSA-B indices between patients with schizophrenia and HCs.

	SZ (N = 163)		HC (N = 620)		Statistics		
	Mean	SD	Mean	SD	Effect size, *d*	*t* or χ^2	*p*
WAIS-III							
Full-Scale IQ	88.1	17.9	112.0	12.1	-1.57	*t* (781) = 20.12	7.3×10^{-73}
Verbal IQ	91.8	17.3	113.0	13.2	-1.38	*t* (781) = 17.04	1.4×10^{-55}
Performance IQ	85.8	18.0	108.3	12.4	-1.46	*t* (781) = 18.60	3.4×10^{-64}
Verbal Comprehension Index	94.0	16.5	110.7	13.1	-1.12	*t* (781) = 13.62	4.7×10^{-38}
Perceptual Organization Index	89.8	18.9	107.9	13.2	-1.11	*t* (781) = 14.10	2.3×10^{-40}
Working Memory Index	90.2	17.9	109.6	15.1	-1.17	*t* (781) = 14.06	3.3×10^{-40}
Processing Speed Index	80.3	16.1	109.8	13.8	-1.96	*t* (781) = 23.37	5.1×10^{-92}
Vocabulary	8.9	3.5	12.4	3.1	-1.08	*t* (781) = 12.68	1.2×10^{-33}
Similarities	8.8	3.1	11.8	2.4	-1.08	*t* (781) = 13.24	3.0×10^{-36}
Information	9.1	3.1	11.5	2.7	-0.82	*t* (781) = 9.73	3.3×10^{-21}
Comprehension	7.7	3.5	12.8	2.7	-1.65	*t* (781) = 20.39	2.1×10^{-74}
Arithmetic	8.3	3.3	12.4	3.0	-1.33	*t* (781) = 15.55	1.1×10^{-47}
Digit Span	9.6	3.3	11.2	3.1	-0.51	*t* (781) = 5.97	3.6×10^{-9}
Letter-Number Sequencing	7.8	3.4	11.4	3.2	-1.06	*t* (781) = 12.29	7.0×10^{-32}
Picture Arrangement	7.9	3.9	10.6	3.2	-0.75	*t* (781) = 9.02	1.4×10^{-18}
Picture Completion	8.0	3.5	10.5	2.6	-0.81	*t* (781) = 10.07	1.6×10^{-22}
Block Design	8.8	4.0	11.8	3.1	-0.83	*t* (781) = 10.19	5.6×10^{-23}
Matrix Reasoning	8.5	3.5	11.7	2.6	-1.04	*t* (781) = 13.00	4.3×10^{-35}
Digit Symbol-Coding	6.5	3.0	11.8	3.0	-1.79	*t* (781) = 20.44	1.0×10^{-74}
Symbol Search	6.5	3.2	11.9	2.8	-1.79	*t* (781) = 21.19	4.4×10^{-79}
UPSA-B							
Finance[a]	43.6	8.3	48.9	2.7	-0.85	*t* (217) = 7.00	3.1×10^{-11}
Communication[a]	24.2	10.9	34.1	6.8	-1.09	*t* (217) = 8.18	2.4×10^{-14}
Total score of the UPSA-B[a]	67.8	16.8	82.9	7.8	-1.16	*t* (217) = 9.14	4.6×10^{-17}

Legend: [a]Only 68 patients with schizophrenia and 151 HCs have the UPSA-B data; *Italic type* means cognitive indices of interest or social indices of interest; Underlining indicates $p < 0.05$.

Abbreviation: SZ, patients with schizophrenia; HCs, healthy controls; SD, standard deviation; WAIS-III, Wechsler Adult Intelligence Scale-Third Edition; IQ, intelligence quotient; UPSA-B, the University of California, San Diego, Performance-Based Skills Assessment Brief.

Supplementary Table 2 Correlations between the MRI indices and the WAIS-III/UPSA-B indices in patients with schizophrenia.

	Lhippo		Rhippo		Lamyg		Ramyg	
	r	*p*	*r*	*p*	*r*	*p*	*r*	*p*
WAIS-III								
Full-Scale IQ	0.301	9.6×10^{-5}	0.301	9.4×10^{-5}	0.293	1.5×10^{-4}	0.214	6.0×10^{-3}
Verbal IQ	0.291	1.7×10^{-4}	0.252	1.1×10^{-3}	0.291	1.6×10^{-4}	0.195	1.3×10^{-2}
Performance IQ	0.272	4.4×10^{-4}	0.318	3.5×10^{-5}	0.253	1.1×10^{-3}	0.206	8.4×10^{-3}
Verbal Comprehension Index	0.314	4.3×10^{-5}	0.275	3.8×10^{-4}	0.330	1.7×10^{-5}	0.216	5.6×10^{-3}
Perceptual Organization Index	0.274	4.1×10^{-4}	0.299	1.1×10^{-4}	0.235	2.5×10^{-3}	0.176	2.4×10^{-2}
Working Memory Index	0.219	5.0×10^{-3}	0.207	8.1×10^{-3}	0.207	7.9×10^{-3}	0.093	0.24
Processing Speed Index	0.252	1.2×10^{-3}	0.252	1.2×10^{-3}	0.166	3.4×10^{-2}	0.173	2.7×10^{-2}
Vocabulary	0.257	9.5×10^{-4}	0.207	8.0×10^{-3}	0.309	5.9×10^{-5}	0.249	1.4×10^{-3}
Similarities	0.255	1.0×10^{-3}	0.220	4.8×10^{-3}	0.311	5.3×10^{-5}	0.172	2.8×10^{-2}
Information	0.291	1.6×10^{-4}	0.273	4.1×10^{-4}	0.238	2.2×10^{-3}	0.160	4.1×10^{-2}
Comprehension	0.230	3.1×10^{-3}	0.216	5.6×10^{-3}	0.230	3.2×10^{-3}	0.190	1.5×10^{-2}
Arithmetic	0.187	1.7×10^{-2}	0.149	5.7×10^{-2}	0.166	3.5×10^{-2}	0.033	0.67
Digit Span	0.201	1.0×10^{-2}	0.166	3.4×10^{-2}	0.165	3.6×10^{-2}	0.126	0.11
Letter-Number Sequencing	0.155	4.9×10^{-2}	0.192	1.4×10^{-2}	0.198	1.1×10^{-2}	0.090	0.25
Picture Arrangement	0.175	2.6×10^{-2}	0.265	6.3×10^{-4}	0.220	4.8×10^{-3}	0.150	5.6×10^{-2}
Picture Completion	0.264	6.5×10^{-4}	0.275	3.8×10^{-4}	0.266	6.0×10^{-4}	0.165	3.5×10^{-2}
Block Design	0.212	6.5×10^{-3}	0.263	6.8×10^{-4}	0.137	8.1×10^{-2}	0.171	2.9×10^{-2}
Matrix Reasoning	0.228	3.4×10^{-3}	0.220	4.8×10^{-3}	0.209	7.4×10^{-3}	0.116	0.14
Digit Symbol-Coding	0.211	6.9×10^{-3}	0.221	4.6×10^{-3}	0.177	2.4×10^{-2}	0.185	1.8×10^{-2}
Symbol Search	0.245	1.6×10^{-3}	0.243	1.8×10^{-3}	0.122	0.12	0.131	9.6×10^{-2}
UPSA-B								
Finance	0.229	6.0×10^{-2}	0.245	4.4×10^{-2}	0.222	6.8×10^{-2}	-0.006	0.96
Communication	0.206	9.3×10^{-2}	0.311	9.9×10^{-3}	0.156	0.20	0.084	0.50
Total score	0.247	4.3×10^{-2}	0.323	7.2×10^{-3}	0.211	8.4×10^{-2}	0.051	0.68

(Continued) **Supplementary Table 2** Correlations between the MRI indices and the WAIS-III/UPSA-B indices in patients with schizophrenia.

	Lthal		Rthal		Laccumb		Raccumb	
	r	*p*	*r*	*p*	*r*	*p*	*r*	*p*
WAIS-III								
Full-Scale IQ	0.136	8.4×10^{-2}	0.299	1.0×10^{-4}	0.179	2.2×10^{-2}	0.267	5.6×10^{-4}
Verbal IQ	0.159	4.3×10^{-2}	0.300	1.0×10^{-4}	0.160	4.2×10^{-2}	0.265	6.4×10^{-4}
Performance IQ	0.095	0.23	0.254	1.1×10^{-3}	0.181	2.0×10^{-2}	0.229	3.2×10^{-3}
Verbal Comprehension Index	0.157	4.5×10^{-2}	0.281	2.8×10^{-4}	0.156	4.7×10^{-2}	0.251	1.3×10^{-3}
Perceptual Organization Index	0.088	0.26	0.192	1.4×10^{-2}	0.145	6.5×10^{-2}	0.201	1.0×10^{-2}
Working Memory Index	0.144	6.7×10^{-2}	0.247	1.5×10^{-3}	0.086	0.27	0.210	7.2×10^{-3}
Processing Speed Index	0.100	0.20	0.261	7.6×10^{-4}	0.16	4.1×10^{-2}	0.244	1.7×10^{-3}
Vocabulary	0.127	0.11	0.262	7.3×10^{-4}	0.144	6.6×10^{-2}	0.238	2.2×10^{-3}
Similarities	0.136	8.4×10^{-2}	0.26	8.1×10^{-4}	0.121	0.12	0.208	7.7×10^{-3}
Information	0.134	8.9×10^{-2}	0.208	7.6×10^{-3}	0.144	6.6×10^{-2}	0.210	7.1×10^{-3}
Comprehension	0.137	8.1×10^{-2}	0.296	1.3×10^{-4}	0.165	3.6×10^{-2}	0.209	7.4×10^{-3}
Arithmetic	0.094	0.24	0.214	6.1×10^{-3}	0.128	0.10	0.161	3.9×10^{-2}
Digit Span	0.135	8.6×10^{-2}	0.218	5.1×10^{-3}	0.050	0.52	0.243	1.8×10^{-3}
Letter-Number Sequencing	0.109	0.17	0.162	3.9×10^{-2}	0.031	0.70	0.140	7.5×10^{-2}
Picture Arrangement	0.091	0.25	0.297	1.2×10^{-4}	0.144	6.7×10^{-2}	0.131	9.7×10^{-2}
Picture Completion	0.076	0.33	0.147	6.1×10^{-2}	0.177	2.4×10^{-2}	0.183	1.9×10^{-2}
Block Design	0.073	0.35	0.132	9.2×10^{-2}	0.086	0.27	0.172	2.8×10^{-2}
Matrix Reasoning	0.084	0.29	0.214	6.0×10^{-3}	0.117	0.14	0.163	3.7×10^{-2}
Digit Symbol-Coding	0.051	0.51	0.219	5.0×10^{-3}	0.179	2.2×10^{-2}	0.266	6.0×10^{-4}
Symbol Search	0.123	0.12	0.256	9.6×10^{-4}	0.121	0.12	0.188	1.6×10^{-2}
UPSA-B								
Finance	0.188	0.12	0.345	4.0×10^{-3}	0.277	2.2×10^{-2}	0.206	9.2×10^{-2}
Communication	0.184	0.13	0.434	2.2×10^{-4}	0.184	0.13	0.230	5.9×10^{-2}
Total score	0.212	8.2×10^{-2}	0.452	1.1×10^{-4}	0.256	3.5×10^{-2}	0.251	3.9×10^{-2}

(Continued) **Supplementary Table 2** Correlations between the MRI indices and the WAIS-III/UPSA-B indices in patients with schizophrenia.

	Lcaud		Rcaud		Lput		Rput	
	r	*p*	*r*	*p*	*r*	*p*	*r*	*p*
WAIS-III								
Full-Scale IQ	0.134	8.8×10^{-2}	0.060	0.45	0.171	2.9×10^{-2}	0.183	1.9×10^{-2}
Verbal IQ	0.156	4.6×10^{-2}	0.060	0.45	0.177	2.4×10^{-2}	0.180	2.2×10^{-2}
Performance IQ	0.091	0.25	0.059	0.46	0.141	7.2×10^{-2}	0.160	4.2×10^{-2}
Verbal Comprehension Index	0.188	1.6×10^{-2}	0.094	0.23	0.182	2.0×10^{-2}	0.152	5.2×10^{-2}
Perceptual Organization Index	0.088	0.27	0.048	0.54	0.115	0.14	0.146	6.4×10^{-2}
Working Memory Index	0.022	0.78	-0.052	0.51	0.119	0.13	0.139	7.7×10^{-2}
Processing Speed Index	0.014	0.86	-0.014	0.86	0.114	0.15	0.128	0.10
Vocabulary	0.229	3.3×10^{-3}	0.128	0.10	0.183	1.9×10^{-2}	0.147	6.0×10^{-2}
Similarities	0.098	0.21	0.045	0.57	0.114	0.15	0.096	0.22
Information	0.159	4.3×10^{-2}	0.063	0.42	0.163	3.7×10^{-2}	0.138	8.0×10^{-2}
Comprehension	0.172	2.8×10^{-2}	0.089	0.26	0.204	9.2×10^{-3}	0.230	3.2×10^{-3}
Arithmetic	0.093	0.24	0.002	0.98	0.111	0.16	0.119	0.13
Digit Span	0.002	0.98	-0.047	0.55	0.068	0.39	0.120	0.13
Letter-Number Sequencing	-0.051	0.52	-0.094	0.23	0.104	0.19	0.109	0.16
Picture Arrangement	0.023	0.78	0.024	0.76	0.109	0.17	0.099	0.21
Picture Completion	0.080	0.31	0.052	0.51	0.097	0.22	0.125	0.11
Block Design	0.061	0.44	0.009	0.91	0.095	0.23	0.091	0.25
Matrix Reasoning	0.08	0.31	0.068	0.39	0.105	0.18	0.160	4.2×10^{-2}
Digit Symbol-Coding	0.136	8.4×10^{-2}	0.096	0.22	0.168	3.2×10^{-2}	0.162	3.9×10^{-2}
Symbol Search	-0.114	0.15	-0.126	0.11	0.046	0.56	0.068	0.39
UPSA-B								
Finance	0.141	0.25	0.093	0.45	0.158	0.20	0.188	0.12
Communication	0.171	0.16	0.166	0.18	0.086	0.48	0.161	0.19
Total score	0.181	0.14	0.154	0.21	0.134	0.28	0.197	0.11

(Continued) **Supplementary Table 2** Correlations between the MRI indices and the WAIS-III/UPSA-B indices in patients with schizophrenia.

	Lpal		Rpal	
	r	*p*	*r*	*p*
WAIS-III				
Full-Scale IQ	-0.037	0.64	-0.036	0.65
Verbal IQ	0.014	0.86	0.002	0.98
Performance IQ	-0.085	0.28	-0.070	0.37
Verbal Comprehension Index	0.049	0.54	0.015	0.85
Perceptual Organization Index	-0.077	0.33	-0.064	0.42
Working Memory Index	-0.031	0.70	0.001	0.99
Processing Speed Index	-0.048	0.54	0.007	0.93
Vocabulary	0.072	0.36	0.012	0.88
Similarities	-0.019	0.81	-0.034	0.67
Information	0.037	0.64	0.026	0.74
Comprehension	-0.001	0.99	0.019	0.81
Arithmetic	-0.003	0.97	-0.053	0.50
Digit Span	-0.022	0.78	0.037	0.64
Letter-Number Sequencing	-0.058	0.47	-0.004	0.96
Picture Arrangement	-0.104	0.19	-0.117	0.14
Picture Completion	0.011	0.89	0.023	0.77
Block Design	-0.101	0.20	-0.125	0.11
Matrix Reasoning	-0.083	0.29	-0.036	0.65
Digit Symbol-Coding	-0.003	0.97	0.055	0.49
Symbol Search	-0.088	0.26	-0.034	0.66
UPSA-B				
Finance	0.094	0.45	0.168	0.17
Communication	-0.049	0.69	0.125	0.31
Total score	0.014	0.91	0.164	0.18

Legend: *Italic type* means cognitive indices of interest or social indices of interest; Underlining indicates $p < 1.79 \times 10^{-3}$ (0.05/28).

Abbreviations: IQ, intelligence quotient; WAIS-III, Wechsler Adult Intelligence Scale-Third Edition; UPSA-B, the University of California, San Diego, Performance-Based Skills Assessment Brief; Lhippo, left hippocampus; Rhippo, right hippocampus; Laccumb, left accumbens; Raccumb, right accumbens; Lthal, left thalamus; Rthal, right thalamus; Lamyg, left amygdala; Ramyg, right amygdala; Lcaud, left caudate; Rcaud, right caudate; Lput, left putamen; Rput, right putamen; Lpal, left pallidum; Rpal, right pallidum.

Supplementary Table 3 Correlations between the MRI indices and the WAIS-III/UPSA-B indices in HCs.

	Lhippo		Rhippo		Lamyg		Ramyg	
	r	*p*	*r*	*p*	*r*	*p*	*r*	*p*
WAIS-III								
Full-Scale IQ	0.043	0.29	0.040	0.32	0.032	0.42	0.047	0.24
Verbal IQ	0.045	0.26	0.033	0.41	0.033	0.41	0.054	0.18
Performance IQ	0.017	0.68	0.031	0.44	0.020	0.62	0.015	0.72
Verbal Comprehension Index	0.055	0.17	0.043	0.29	0.039	0.34	0.078	5.1×10^{-2}
Perceptual Organization Index	0.016	0.68	0.044	0.28	0.010	0.80	0.003	0.94
Working Memory Index	0.010	0.80	-0.009	0.81	0.042	0.29	-0.004	0.92
Processing Speed Index	-0.023	0.57	-0.046	0.25	-0.006	0.87	0.025	0.54
Vocabulary	0.052	0.20	0.044	0.27	0.059	0.14	0.055	0.17
Similarities	0.092	0.02	0.057	0.16	0.041	0.31	0.069	8.7×10^{-2}
Information	0.010	0.81	0.012	0.77	-0.009	0.82	0.079	5.0×10^{-2}
Comprehension	0.006	0.87	-0.007	0.86	-0.008	0.84	0.028	0.48
Arithmetic	0.006	0.89	0.005	0.89	0.014	0.73	0.030	0.45
Digit Span	0.039	0.33	0.040	0.32	0.052	0.20	-0.014	0.73
Letter-Number Sequencing	-0.014	0.73	-0.063	0.12	0.041	0.30	-0.014	0.74
Picture Arrangement	0.012	0.77	0.021	0.60	0.030	0.46	-0.005	0.90
Picture Completion	0.020	0.62	0.055	0.17	0.001	0.98	0.007	0.87
Block Design	0.020	0.63	0.042	0.30	0.003	0.95	0.005	0.89
Matrix Reasoning	-0.005	0.89	-0.003	0.95	0.018	0.66	-0.004	0.91
Digit Symbol-Coding	0.012	0.77	-0.016	0.68	0.013	0.75	0.048	0.23
Symbol Search	-0.053	0.19	-0.064	0.11	-0.028	0.49	-0.008	0.84
UPSA-B								
Finance	-0.060	0.47	-0.035	0.67	-0.112	0.17	-0.112	0.17
Communication	-0.036	0.66	-0.018	0.83	-0.006	0.94	0.014	0.86
Total score	-0.052	0.53	-0.028	0.73	-0.044	0.59	-0.026	0.75

(Continued) **Supplementary Table 3** Correlations between the MRI indices and the WAIS-III/UPSA-B indices in HCs.

	Lthal		Rthal		Laccumb		Raccumb	
	r	*p*	*r*	*p*	*r*	*p*	*r*	*p*
WAIS-III								
Full-Scale IQ	0.092	2.2×10^{-2}	0.091	2.4×10^{-2}	0.026	0.53	0.083	4.0×10^{-2}
Verbal IQ	0.082	4.2×10^{-2}	0.061	0.13	0.019	0.63	0.082	4.1×10^{-2}
Performance IQ	0.067	9.6×10^{-2}	0.092	2.2×10^{-2}	0.021	0.61	0.060	0.13
Verbal Comprehension Index	0.079	4.9×10^{-2}	0.068	9.3×10^{-2}	0.026	0.53	0.086	3.3×10^{-2}
Perceptual Organization Index	0.043	0.28	0.079	4.8×10^{-2}	0.006	0.89	0.051	0.20
Working Memory Index	0.040	0.32	0.067	9.8×10^{-2}	0.006	0.89	0.037	0.36
Processing Speed Index	0.040	0.32	0.031	0.44	0.025	0.54	0.015	0.72
Vocabulary	0.092	2.2×10^{-2}	0.070	8.0×10^{-2}	0.029	0.47	0.061	0.13
Similarities	0.073	6.9×10^{-2}	0.081	4.4×10^{-2}	0.025	0.53	0.090	2.5×10^{-2}
Information	0.038	0.35	0.028	0.48	0.020	0.62	0.072	7.4×10^{-2}
Comprehension	0.073	6.7×10^{-2}	0.012	0.76	-0.010	0.80	0.076	6.0×10^{-2}
Arithmetic	0.027	0.50	0.028	0.48	0.013	0.74	0.024	0.55
Digit Span	0.054	0.18	0.061	0.13	0.011	0.78	0.043	0.29
Letter-Number Sequencing	0.017	0.67	0.076	5.9×10^{-2}	-0.013	0.75	0.012	0.77
Picture Arrangement	0.022	0.59	0.052	0.19	0.029	0.46	0.033	0.41
Picture Completion	0.028	0.48	0.081	4.3×10^{-2}	0.045	0.26	0.032	0.43
Block Design	0.016	0.70	0.062	0.12	-0.016	0.69	0.026	0.52
Matrix Reasoning	0.057	0.16	0.031	0.44	-0.015	0.71	0.051	0.20
Digit Symbol-Coding	0.087	3.1×10^{-2}	0.054	0.18	0.019	0.64	0.039	0.33
Symbol Search	-0.019	0.65	0.002	0.97	0.026	0.52	-0.010	0.81
UPSA-B								
Finance	-0.047	0.57	-0.075	0.36	-0.002	0.98	-0.007	0.93
Communication	0.014	0.86	-0.013	0.88	0.057	0.49	-0.042	0.61
Total score	-0.004	0.96	-0.037	0.65	0.049	0.55	-0.039	0.63

(Continued) **Supplementary Table 3** Correlations between the MRI indices and the WAIS-III/UPSA-B indices in HCs.

	Lcaud		Rcaud		Lput		Rput	
	r	*p*	*r*	*p*	*r*	*p*	*r*	*p*
WAIS-III								
Full-Scale IQ	0.036	0.37	0.024	0.55	-0.010	0.81	0.006	0.88
Verbal IQ	0.052	0.19	0.059	0.14	-0.006	0.89	0.025	0.54
Performance IQ	0.003	0.95	-0.031	0.44	-0.001	0.99	-0.015	0.70
Verbal Comprehension Index	0.070	8.1×10^{-2}	0.069	8.5×10^{-2}	-0.005	0.90	0.025	0.54
Perceptual Organization Index	0.022	0.58	0.011	0.78	0.010	0.80	0.004	0.92
Working Memory Index	-0.002	0.96	-0.022	0.59	0.023	0.57	0.030	0.46
Processing Speed Index	0.003	0.94	-0.049	0.22	0.009	0.83	0.018	0.66
Vocabulary	0.081	4.3×10^{-2}	0.065	0.10	-0.007	0.87	0.032	0.42
Similarities	0.066	0.10	0.082	4.2×10^{-2}	0.038	0.34	0.035	0.39
Information	0.027	0.49	0.030	0.45	-0.039	0.34	0.002	0.95
Comprehension	0.035	0.39	0.074	6.7×10^{-2}	-0.028	0.48	0.017	0.66
Arithmetic	0.053	0.19	0.048	0.24	-0.005	0.90	-0.013	0.75
Digit Span	-0.035	0.39	-0.035	0.39	0.019	0.64	0.038	0.34
Letter-Number Sequencing	-0.016	0.68	-0.050	0.21	0.046	0.25	0.052	0.20
Picture Arrangement	-0.039	0.33	-0.077	5.5×10^{-2}	-0.028	0.49	-0.083	3.9×10^{-2}
Picture Completion	0.023	0.57	-0.001	0.97	0.044	0.27	0.016	0.69
Block Design	0.015	0.72	0.019	0.64	0.005	0.90	-0.001	0.98
Matrix Reasoning	0.010	0.81	0.005	0.89	-0.030	0.46	-0.006	0.89
Digit Symbol-Coding	0.011	0.79	-0.030	0.46	0.012	0.77	0.037	0.35
Symbol Search	0.000	1.00	-0.050	0.21	0.004	0.93	-0.002	0.95
UPSA-B								
Finance	0.000	1.00	0.048	0.56	-0.086	0.30	-0.034	0.68
Communication	0.043	0.60	0.038	0.64	0.006	0.94	-0.061	0.45
Total score	0.038	0.65	0.050	0.54	-0.024	0.77	-0.066	0.42

(Continued) **Supplementary Table 3** Correlations between the MRI indices and the WAIS-III/UPSA-B indices in HCs.

	Lpal		Rpal	
	r	*p*	*r*	*p*
WAIS-III				
Full-Scale IQ	-0.002	0.96	0.012	0.77
Verbal IQ	-0.002	0.97	0.028	0.49
Performance IQ	0.005	0.91	-0.007	0.86
Verbal Comprehension Index	0.015	0.70	0.035	0.39
Perceptual Organization Index	0.011	0.78	-0.006	0.89
Working Memory Index	0.021	0.61	0.022	0.58
Processing Speed Index	0.024	0.55	-0.032	0.43
Vocabulary	0.010	0.80	0.030	0.46
Similarities	0.041	0.31	0.038	0.35
Information	-0.001	0.97	0.029	0.47
Comprehension	-0.016	0.70	-0.005	0.90
Arithmetic	-0.011	0.79	0.015	0.70
Digit Span	-0.020	0.62	0.013	0.75
Letter-Number Sequencing	0.074	6.5×10^{-2}	0.018	0.65
Picture Arrangement	-0.035	0.39	0.016	0.70
Picture Completion	0.066	9.9×10^{-2}	-0.011	0.79
Block Design	-0.025	0.54	-0.031	0.44
Matrix Reasoning	-0.015	0.71	0.033	0.41
Digit Symbol-Coding	0.029	0.48	-0.030	0.45
Symbol Search	0.018	0.65	-0.025	0.54
UPSA-B				
Finance	-0.113	0.17	-0.022	0.79
Communication	0.121	0.14	0.035	0.67
Total score	0.067	0.42	0.023	0.78

Legend: *Italic type* means cognitive indices of interest or social indices of interest; there was no significant correlation in HCs [$p < 1.79\times10^{-3}$ (0.05/28)].

Abbreviations: HC, healthy control; IQ, intelligence quotient; WAIS-III, Wechsler Adult Intelligence Scale-Third Edition; UPSA-B, the University of California, San Diego, Performance-Based Skills Assessment Brief; Lhippo, left hippocampus; Rhippo, right hippocampus; Laccumb, left accumbens; Raccumb, right accumbens; Lthal, left thalamus; Rthal, right thalamus; Lamyg, left amygdala; Ramyg, right amygdala; Lcaud, left caudate; Rcaud, right caudate; Lput, left putamen; Rput, right putamen; Lpal, left pallidum; Rpal, right pallidum.

Supplementary figure legends

Supplementary Figure 1 The histograms of the age distribution in schizophrenia group and healthy control group. The unit of age is year.

Supplementary Figure 2 The correlation map among the regional brain volumes in all participants. Abbreviations: L, left; R, right.

Supplementary Figure 3 The correlation map among the regional brain volumes in schizophrenia group. Abbreviations: L, left; R, right.

Supplementary Figure 4 The correlation map among the regional brain volumes in healthy control group. Abbreviations: L, left; R, right.

Supplementary Figure 5 The correlation map among the premorbid IQ and cognitive and social indices in all participants. Abbreviations: IQ, intelligence quotient; UPSA-B, the University of California, San Diego, Performance-Based Skills Assessment Brief.

Supplementary Figure 6 The correlation map among the premorbid IQ and cognitive and social indices in schizophrenia group. Abbreviations: IQ, intelligence quotient; UPSA-B, the University of California, San Diego, Performance-Based Skills Assessment Brief.

Supplementary Figure 7 The correlation map among the premorbid IQ and cognitive

and social indices in healthy control group. Abbreviations: IQ, intelligence quotient; UPSA-B, the University of California, San Diego, Performance-Based Skills Assessment Brief.

Supplementary Figure 8 The Scatter plots between subcortical brain volumes and age in all participants. The unit of subcortical regional volumes is mm^3; The unit of age is year.

Supplementary Figure 9 The Scatter plots between subcortical brain volumes and age in schizophrenia group. The unit of subcortical regional volumes is mm^3; The unit of age is year.

Supplementary Figure 10 The Scatter plots between subcortical brain volumes and age in healthy control group. The unit of subcortical regional volumes is mm^3; The unit of age is year.

Koshiyama *et al.*, Supplementary Figure 1

A Schizophrenia group

B Healthy control group

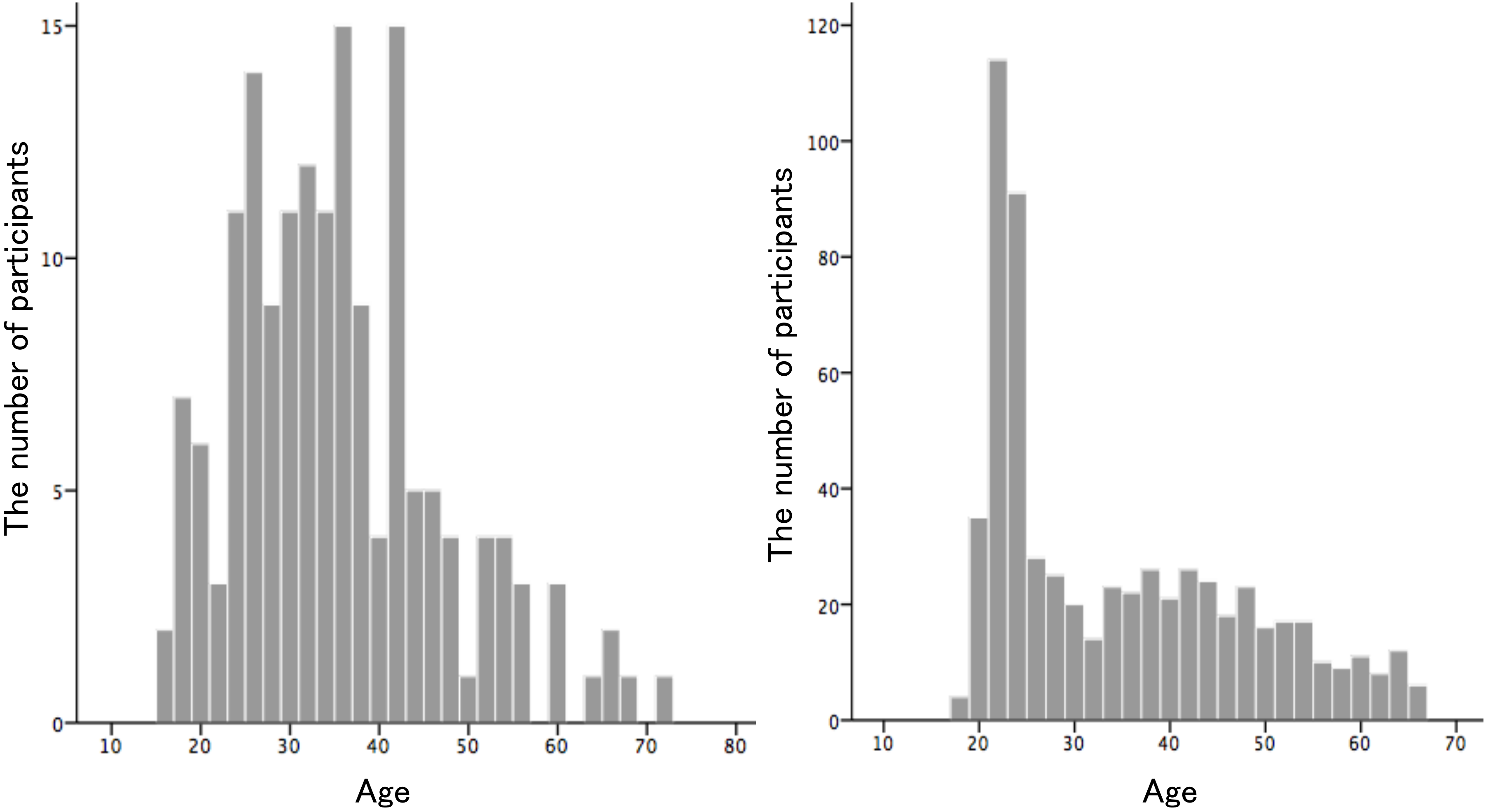

Koshiyama *et al.*, Supplementary Figure 2

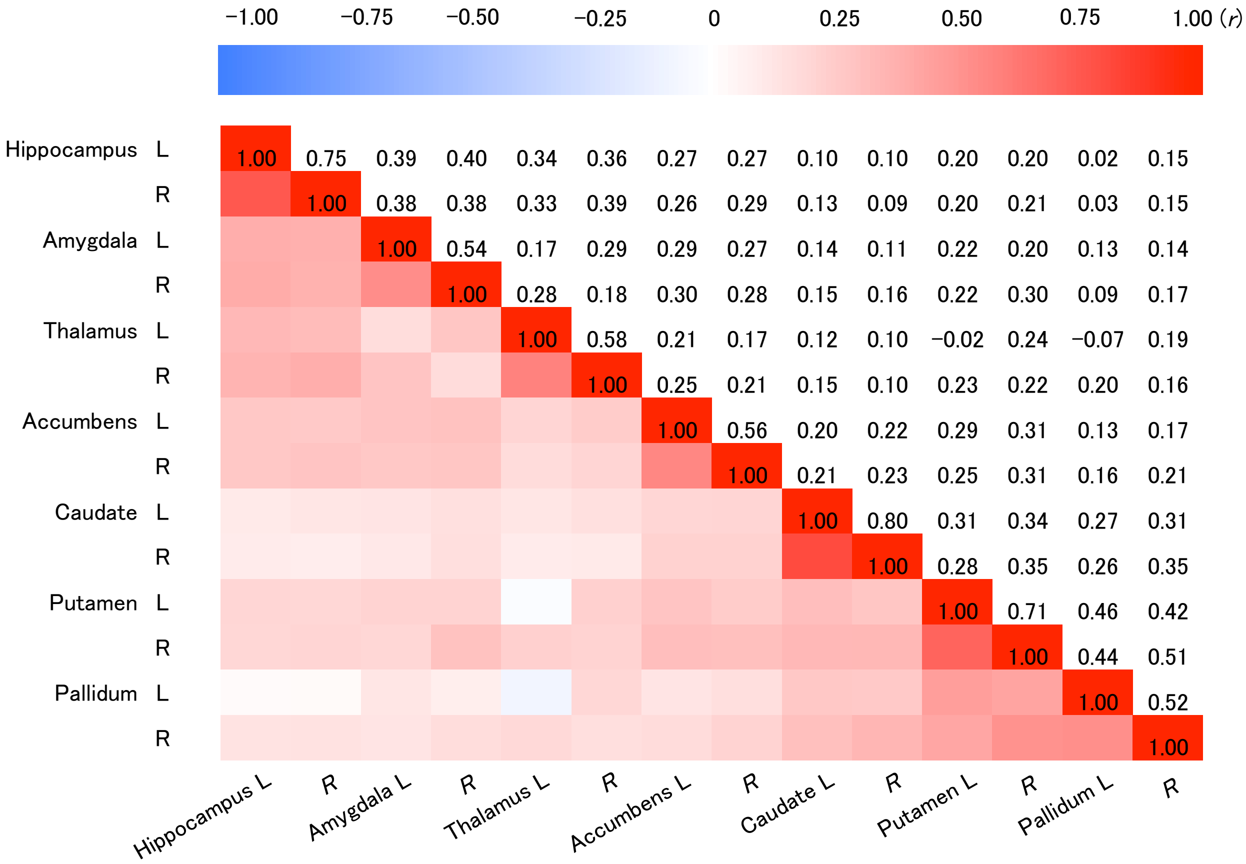

Koshiyama *et al.*, Supplementary Figure 3

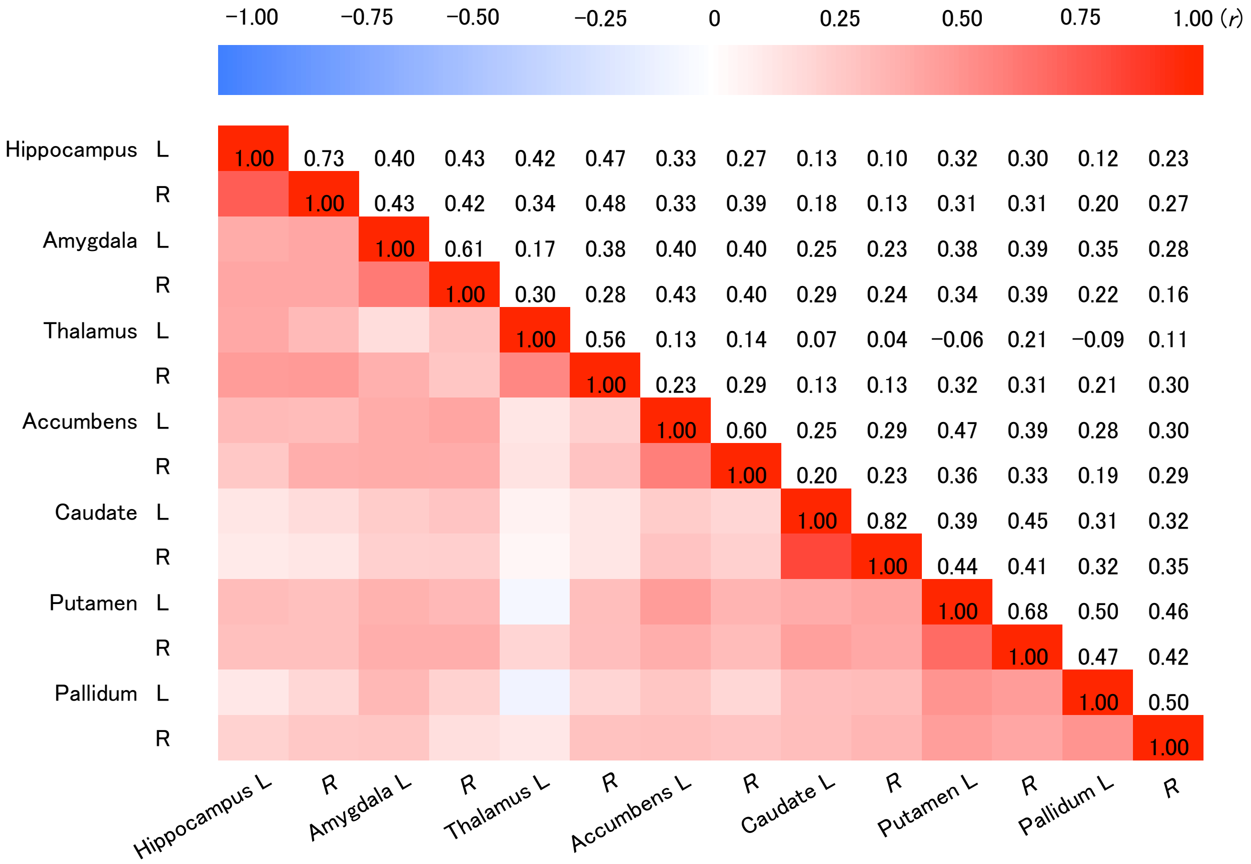

Koshiyama *et al.*, Supplementary Figure 4

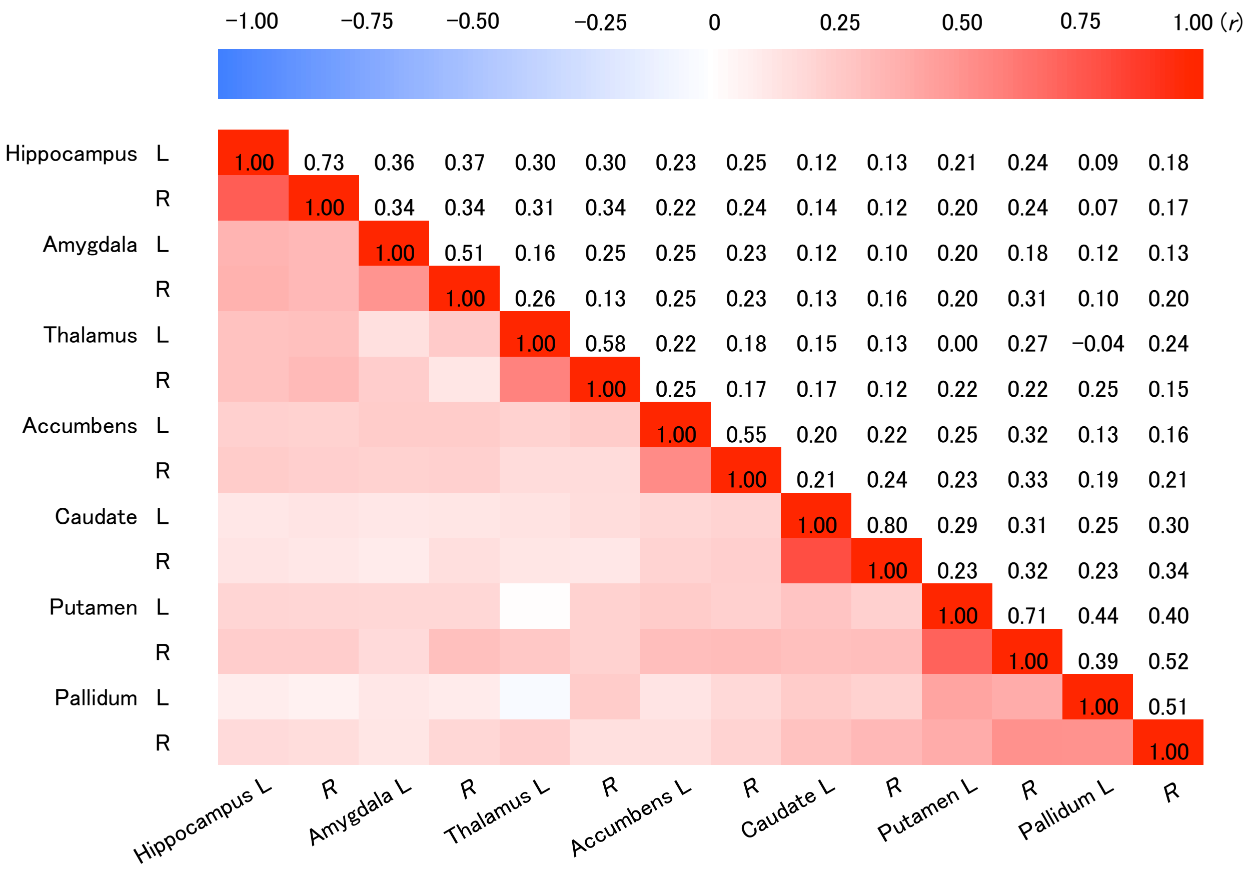

Koshiyama *et al.*, Supplementary Figure 5

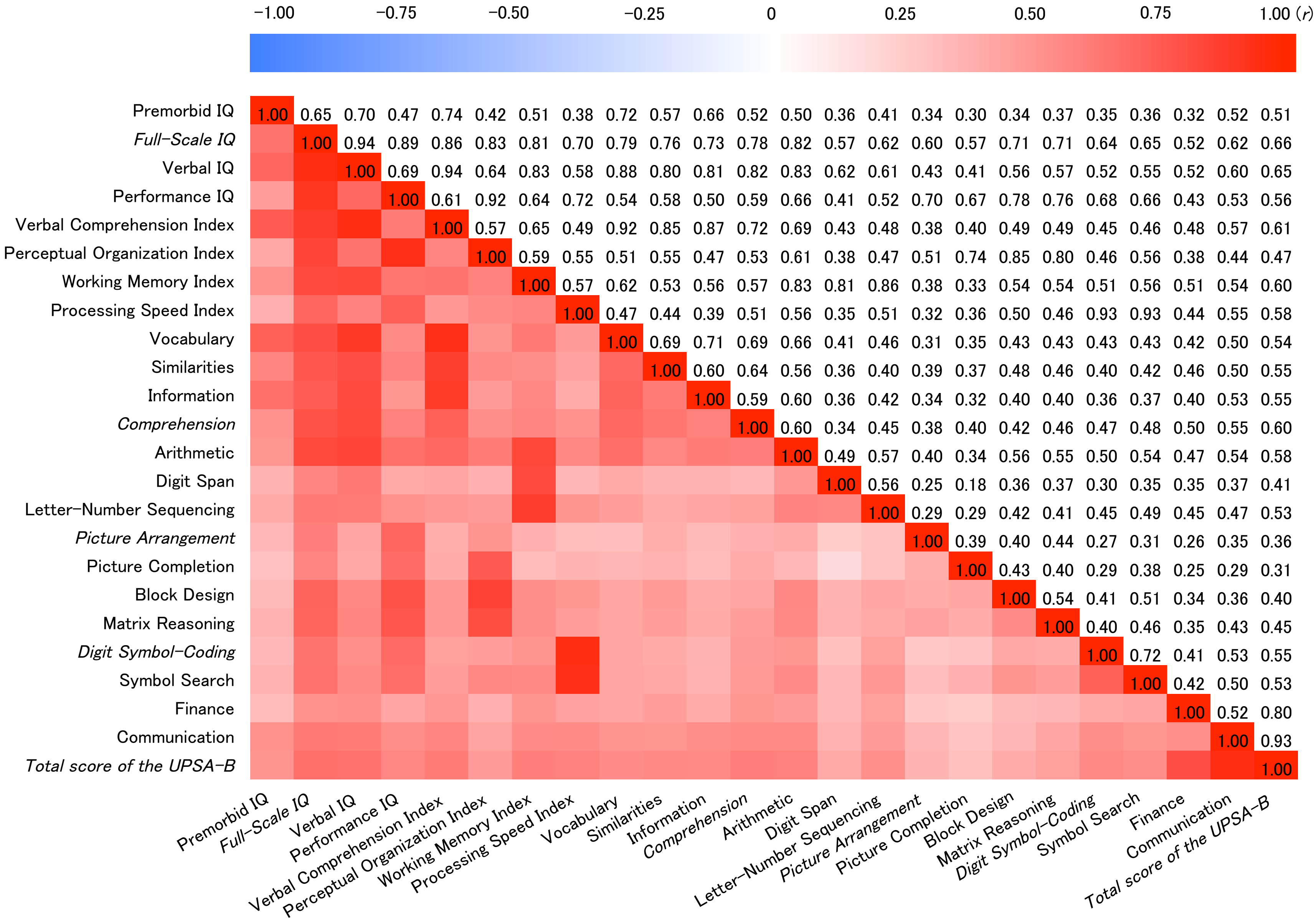

Koshiyama *et al.*, Supplementary Figure 6

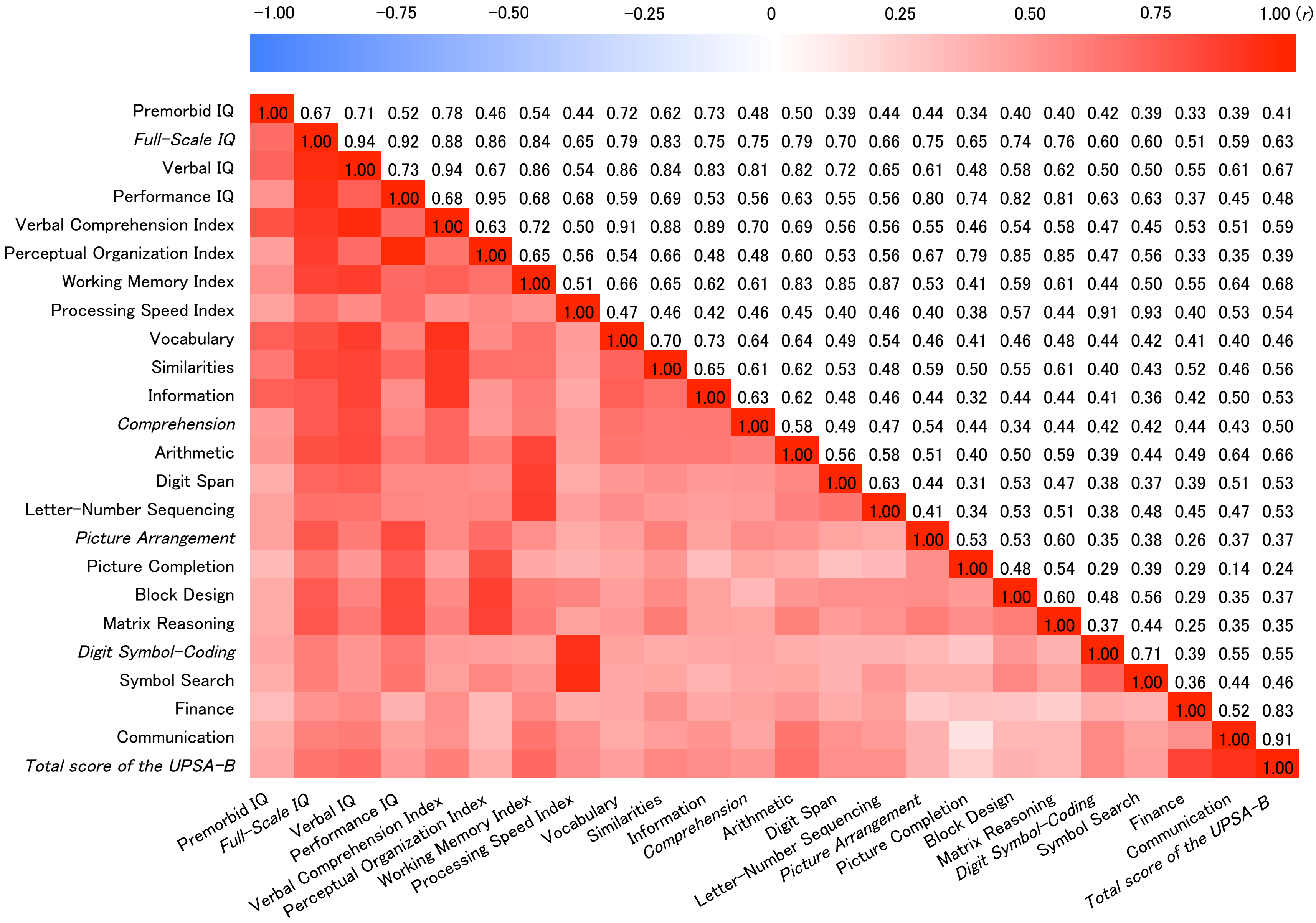

Koshiyama *et al*., Supplementary Figure 7

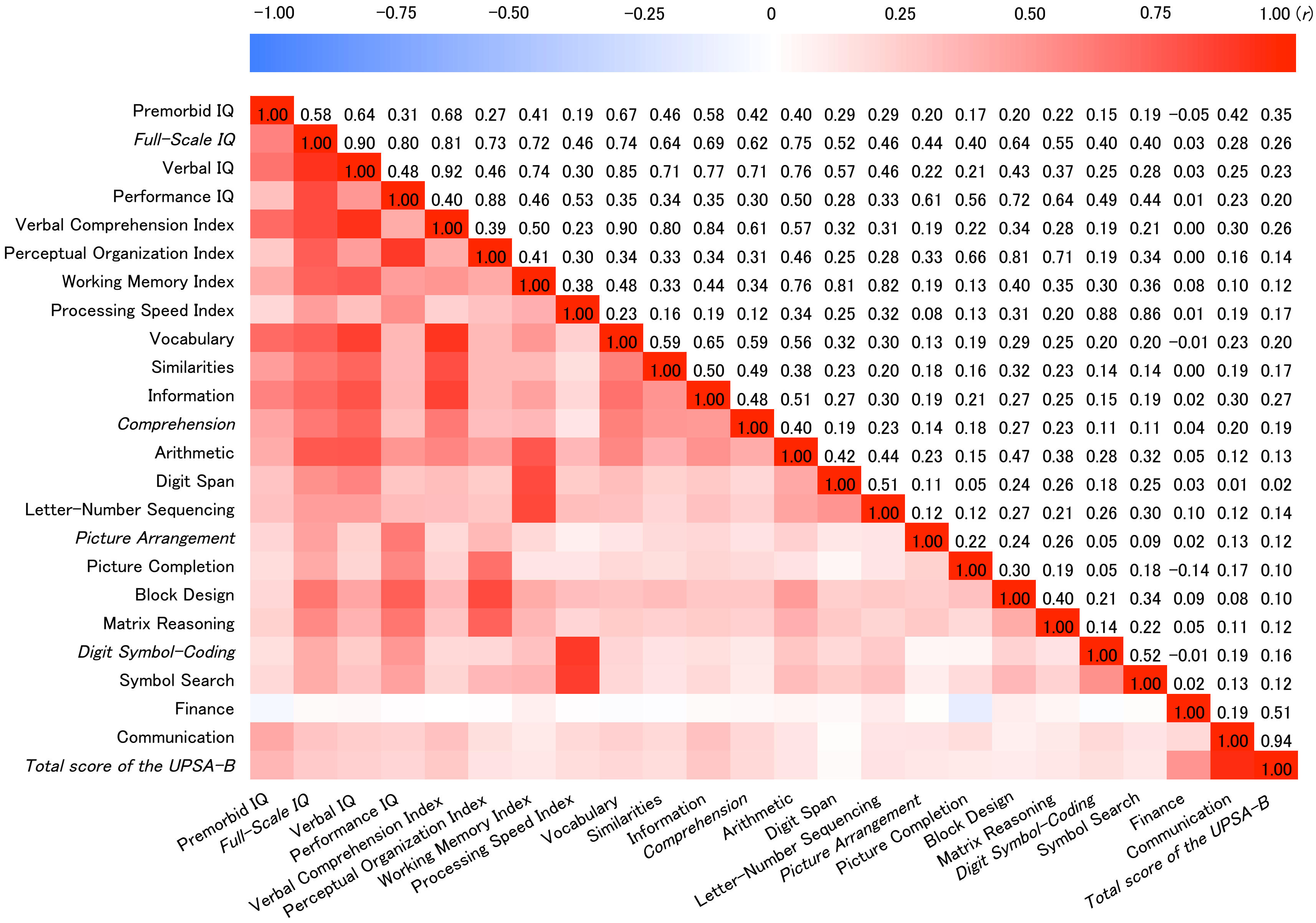

Koshiyama *et al.*, Supplementary Figure 8

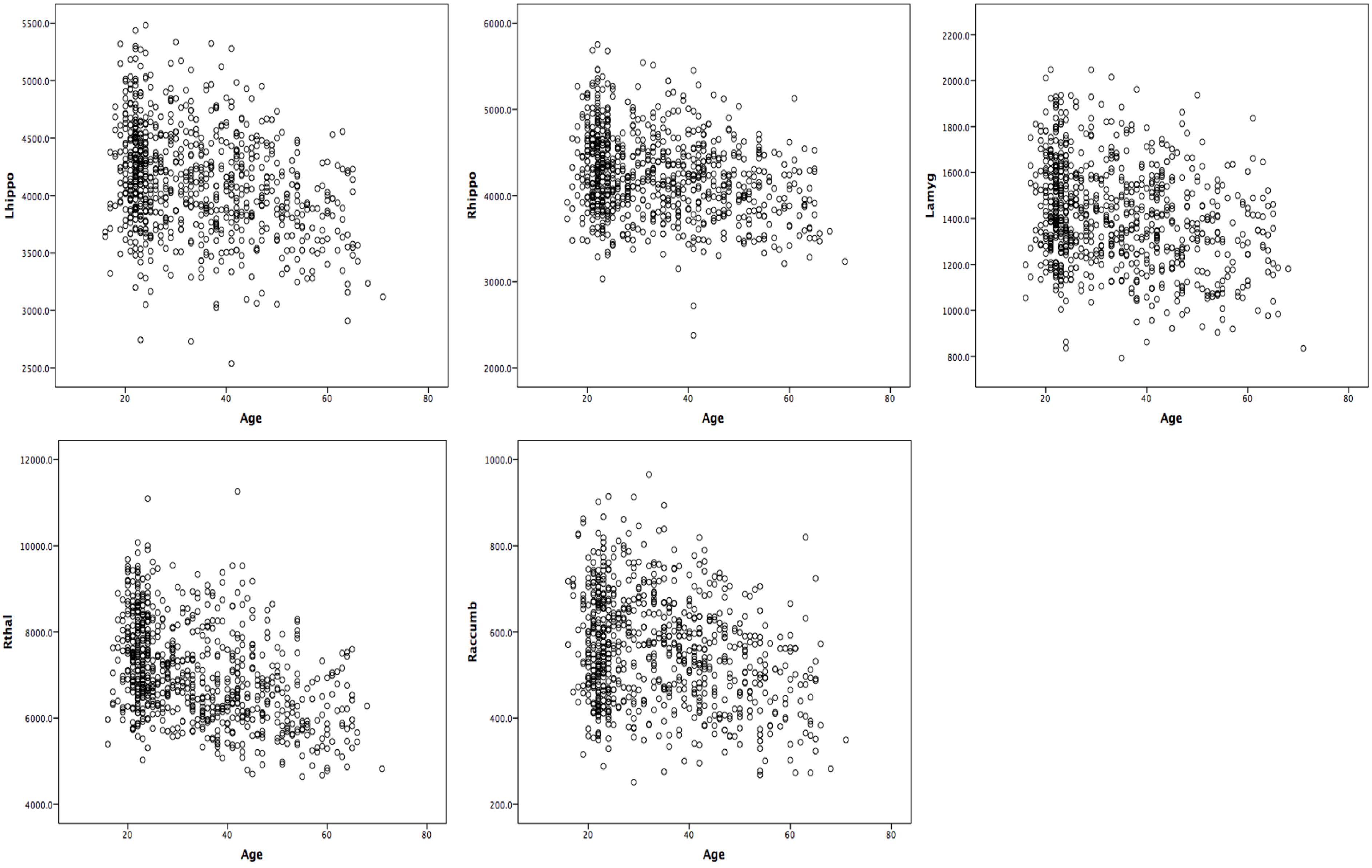

Koshiyama *et al.*, Supplementary Figure 9

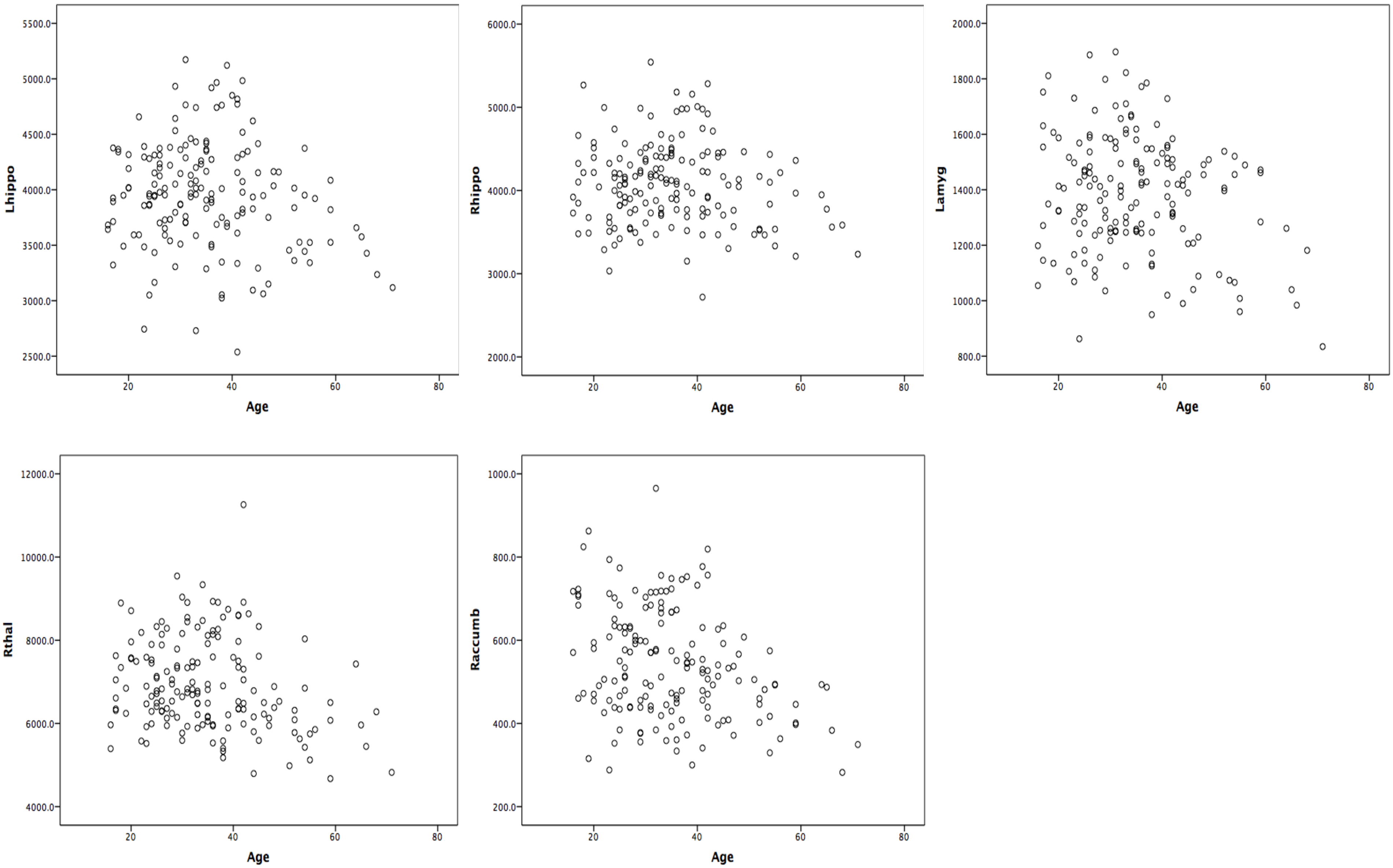

Koshiyama *et al.*, Supplementary Figure 10

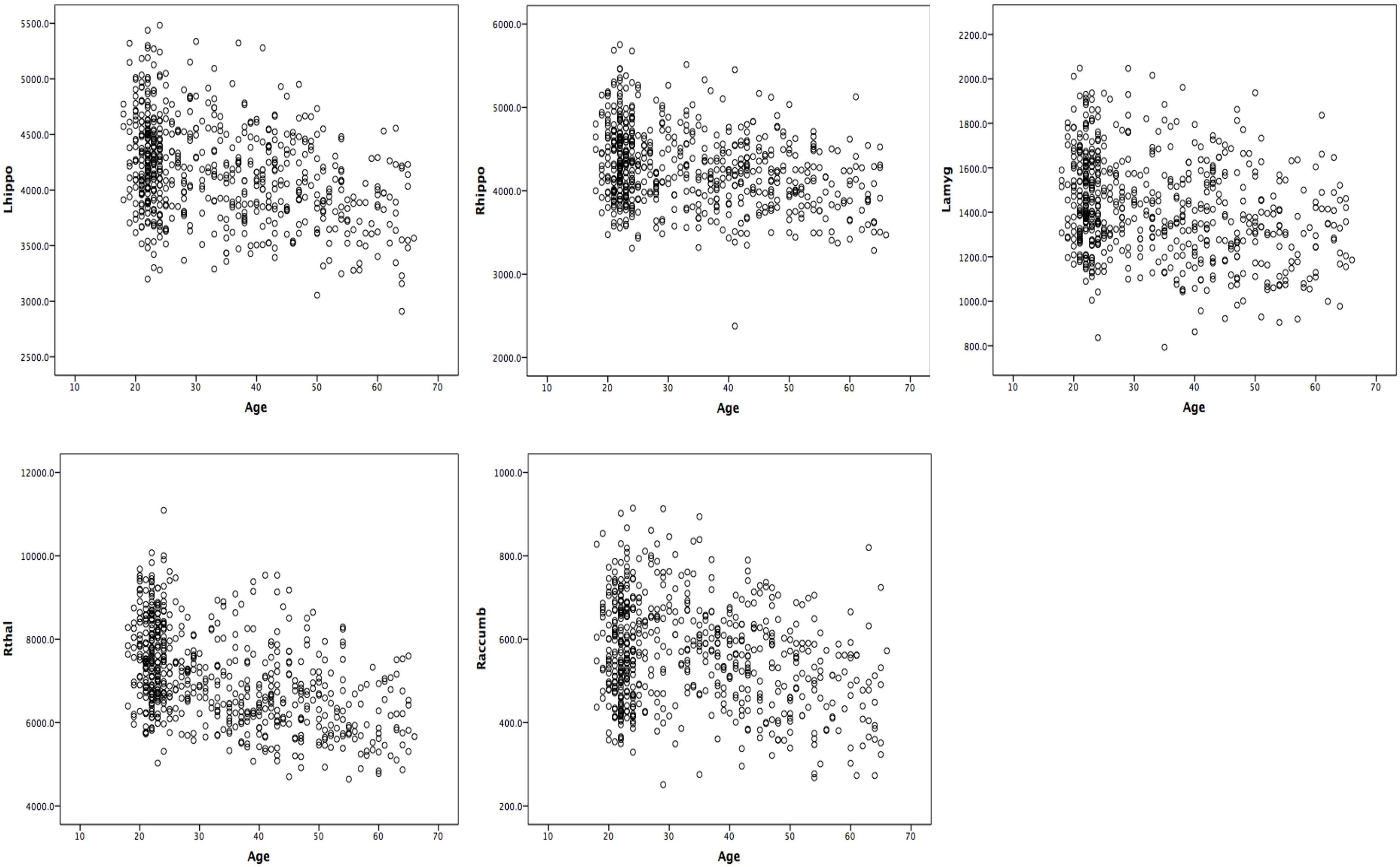

Supplement: Supplementary file 1 — Supplementary Information [file 41598_2017_18950_MOESM1_ESM.pdf]
